# Prevention and Management of Cow’s Milk Allergy in Non-Exclusively Breastfed Infants

**DOI:** 10.3390/nu9070731

**Published:** 2017-07-10

**Authors:** Yvan Vandenplas

**Affiliations:** Kidz Health Castle, UZ Brussel, Vrije Universiteit Brussel, Laarbeeklaan 101, 1090 Brussels, Belgium; yvan.vandenplas@uzbrussel.be; Tel.: +32-2-477-5780; Fax: +32-2-477-5784

**Keywords:** cow milk allergy, hydrolysate, infant formula, functional gastrointestinal disorder, prevention, treatment

## Abstract

**Introduction:** The prevention and management of cow milk allergy (CMA) is still debated. Since CMA is much less frequent in breastfed infants, breastfeeding should be stimulated. **Method:** Literature was searched using databases to find original papers and reviews on this topic. **Results:** Hydrolysates with a clinical proof of efficacy are recommended in the prevention and treatment of CMA. However, not all meta-analyses conclude that hydrolysates do prevent CMA or other atopic manifestations such as atopic dermatitis. There are pros and cons to consider partially hydrolysed protein as an option for starter infant formula for each non-exclusively breastfed infant. A challenge test is still recommended as the most specific and sensitive diagnostic test, although a positive challenge test does not proof that the immune system is involved. The Cow Milk Symptom Score (CoMiSS™) is an awareness tool that enables healthcare professionals to better recognize symptoms related to the ingestion of cow milk, but it still needs validation as diagnostic tool. The current recommended elimination diet is a cow milk based extensive hydrolysate, although rice hydrolysates or soy infant formula can be considered in some cases. About 10 to 15% of infants allergic to cow milk will also react to soy. Mainly because of the higher cost, amino acid based formula is reserved for severe cases. There is no place for infant formula with intact protein from other animals as cross-over allergenicity is high. During recent years, attention focused also on the bifidogenic effect of prebiotics and more recently also on human milk oligosaccharides. A bifidogenic gastrointestinal microbiome may decrease the risk to develop allergic disease. The addition of probiotics and prebiotics to the elimination diet in treatment may enhance the development of tolerance development. **Conclusion:** Breastfeeding is the best way to feed infants. Cow milk based extensive hydrolysates remain the first option for the treatment of CMA for the majority of patients, while amino acid formulas are reserved for the most severe cases. Rice hydrolysates and soy infant formula are second choice options. Partial hydrolysates with clinical proof of efficacy are recommended in some guidelines in the prevention of CMA and allergic disease in at risk infants, and may be considered as an option as protein source in starter infant formula.

## 1. Introduction

This manuscript discusses the prevention and management of cow’s milk allergy (CMA) in non-exclusively breastfed infants. CMA is an adverse health effect arising from a specific immune response that occurs reproducibly on exposure to a protein present in cow milk. Breastfeeding is the first choice feeding for infants, and allergic and functional gastrointestinal disorders occur more often in non-exclusively breastfed than in breastfed infants. The prevalence of allergic diseases involving the gastrointestinal (GI) tract, respiratory tract and the skin is likely to be rising worldwide [1]. Food allergy is a growing health concern in the westernized world with approximately 6% of children suffering from it [2]. CMA is one of the most frequent causes of food allergies in young children with an estimated prevalence between 1.9% and 4.9% in the first year of life [3,4]. Whether there has also been an increase in CMA has not been thoroughly studied [5]. According to a report from Denmark, CMA is up to half of the allergic children immunoglobulin E (IgE) mediated [6]. The risk to develop allergic disease is multifactorial. Recent evidence suggests that low blood vitamin D level is a risk factor for food allergy; vitamin D deficiency predisposes to GI infections, which may promote the development of food allergy. Several data suggest that serum 25-hydroxyvitamin D levels are often insufficient in children with asthma, atopic dermatitis, and food allergy [7]. There is no evidence that supplementation of poly-unsaturated fatty acids in infancy has an effect on infant or childhood allergy, asthma, dermatitis/eczema or food allergy [8]. Many infants present with symptoms related to milk ingestion. The most frequent symptoms and signs related to CMA are listed in Table 1. Both IgE and non-IgE mediated CMA exist. Allergic symptoms must be reproducible. The involvement of the immune system in non-IgE mediated allergy is difficult to demonstrate. Non-IgE mediated allergy is the cause of symptoms in a subset of patients with “hypersensitivity”. Sometimes the symptoms caused by ingestion of milk are very likely to be immune mediated, as in the case of atopic dermatitis improving during a cow milk elimination diet. However, in the case of GI symptoms, such as regurgitation, constipation or general symptoms such as crying or distress, the involvement of the immune system cannot (easily) be demonstrated. . It is likely that there is overlap between the latter and functional GI symptoms. Experts agreed that the likely prevalence for colic, regurgitation, and functional constipation is 20%, 30% and 15%, respectively [9]. The perception of parents that an infant may have cow milk related symptoms is much greater than the reported incidence of CMA since parents report an incidence of up to 17% [10]. The relationship between some of these common symptoms of infancy and CMA is not clear. The best example may be upper respiratory tract symptoms which can seldom be related to CMA, but most frequently are caused by viral infections. It is only in a minority of infants that functional GI symptoms such as regurgitation, constipation and colic are of allergic origin. Intolerance is the consequence of lactase deficiency, the brush border enzyme that digests lactose, the predominant sugar in milk, and is almost always secondary to another condition in young infants. The meaning and definition of a “hypo-allergenic formula” varies in different parts of the world. While in Europe a “hypo-allergenic formula” means a formula that contains hydrolyzed protein and thus a reduced allergenicity, the American Academy of Pediatrics defined it as a formula that is effective in the treatment of at least 90% of the children with CMA, with a 95% confidence interval. It has to be recognized that an extensive hydrolysate is “tolerated” by the vast majority of CMA-patients but that such an elimination diet is not really “treatment” as the elimination diet does not change the immune response. Oral immunotherapy or anti-IgE actually modify the individual propensity to react to cow’s milk and are therefore therapeutic. However, since most literature, including guidelines, recommend the use of extensive hydrolysates as first choice in the management, “treatment” is used in this context.

Tolerance of cow milk will have developed in 85% to 90% of the infants with CMA by the age of three years. High IgE levels predict a longer persistence of allergic reactions to cow milk. In particular, GI symptoms show a good prognosis, suggesting again an overlap between functional GI symptoms and CMA [3,6]. However, most of the information on the natural evolution of CMA comes from tertiary care or specialized centers and only the most severe cases are seen in these centers. This means that data on the natural evolution of CMA at the primary healthcare level are missing.

## 2. Methods

The PubMed and Cochrane Library databases were searched up to July 2016. The searches were limited to human studies and to studies published in English. Only published data were considered.

## 3. Prevention

The allergic march describes the order in which atopic disease develops, starting with atopic dermatitis followed by asthma to end with rhinoconjunctivitis [11]. The development of atopic disease is influenced by environmental and genetic, thus epi-genetic, confounders. 

Two meta-analyses including selected papers on one partial hydrolysate conclude that selected partially and extensive hydrolyzed infant formula may prevent the development of atopic dermatitis and possibly that of CMA [12,13]. Boyle et al concluded in a meta-analysis including much more trials (37 compared to 11 and 15 [14]) that overall there was no consistent evidence that partially or extensively hydrolysed formulas reduce risk of allergic or autoimmune outcomes in infants at high pre-existing risk of these outcomes [14]. Odds ratios for eczema at age 0–4, compared with standard cows’ milk formula, were 0.84 (95% confidence interval 0.67 to 1.07) for partially hydrolysed formula; 0.55 (0.28 to 1.09) for extensively hydrolysed casein based formula; and 1.12 (0.88 to 1.42) for extensively hydrolysed whey based formula [14]. A large study with a negative outcome with a different partial whey hydrolysate than the one included in the above mentioned two meta-analyses contributes largely to these findings [15]. These findings also suggest that outcomes obtained with one hydrolysate may not be extrapolated to another hydrolysate, and that findings are hydrolysate-specific. According to Boyle et al, there is no evidence to support the health claim approved by the US Food and Drug Administration that a partially hydrolysed formula could reduce the risk of eczema nor the conclusion of the Cochrane review that hydrolysed formula could allergy to cows’ milk [14]. This is only partially confirmed by the recent Cochrane review reported that in infants at high risk of allergy not exclusively breast fed, very low-quality evidence suggests that prolonged hydrolysed formula feeding compared with CMF feeding reduces infant allergy and infant CMA ([16] -Cochrane review withdrawn). Studies have found no difference in childhood allergy and no difference in specific allergy, including infant and childhood asthma, eczema and rhinitis and infant food allergy [16]. Although extensively hydrolyzed formulas (eHF) can be used in prevention, they are not considered as first option as they are much more expensive that partially HF (pHF). Because of their bitter taste, eHF have a poor palatability. In theory, the allergenic epitopes are destroyed in the manufacturing process of eHF. pHF has been developed to decrease the amount of epitopes that possibly induce sensitization, while still having peptides of sufficient immunogenicity to induce oral tolerance. Only these pHF can be recommended for prevention for which there are sufficient clinical data to support their efficacy, which are missing for the majority of the commercialized pHFs. Some guidelines recommend the use of pHFs in “at risk” infants, which are defined as infants born in a family in which at least one of the family members (parents, brother, sister) has atopic disease. There is no consensus if this diagnosis of atopic disease should be “doctor confirmed” or not. As a consequence of the ongoing debate some countries (e.g., Japan, UK, Finland, Australia) do not recommend the use of pHF to prevent allergy. There is no place for infant formula with intact protein from different origin in the prevention of allergic disease. Soy protein infant formula has no place in the prevention of atopic disease.

**Figure 1:**
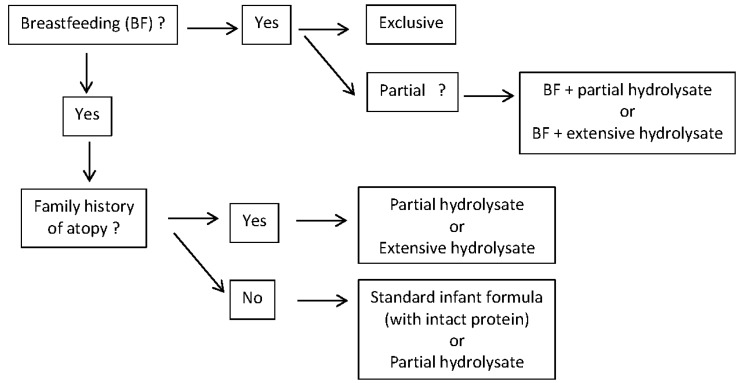
Proposed dietary options according to breastfeeding and/or family history of atopic disease.

Epidemiological data show that about half of the infants that will develop allergy are not part of this “at risk” group [17]. This is due to the fact that although the risk is lower in the non-at risk group, the number of infants in the non-at risk group is much larger. In other words: guidelines recommend today prevention only for half of the infants that will develop atopic disease, and not for the other half. A recent analysis from the 15 year follow-up of the two German birth cohorts GINI-plus and LISA-plus reported for the first time that parental allergic diseases increase the risk of childhood allergic diseases, especially for asthma, independent on whether the first onsets was before or after the birth of a child [18]. Knowledge on the long-term effects of pHF on growth and body composition outcomes in healthy infants later in life is still limited [19]. There are some indications that hydrolysed protein results in metabolic responses more distinctly different from those of human milk and different metabolic organ development compared to intact protein [19,20]. However, FDA and EFSA regulatory authorities consider a partially hydrolysed protein source as a protein source that can be used in starter infant formula, irrespective of the fact if there would be some prevention of allergy or not. All studies with pHF show no or some benefit, but never an increased risk for adverse effects. So the question should be asked is pHF should not be considered as the best second choice infant feeding for every infant, at least for those pHFs with clinical data supporting their efficacy (Figure 1), irrespective of the fact if there is a preventive effect on allergy or not. Opponents to this viewpoint state that breast milk contains intact protein, and a pHF does not. This is true. But: breast milk contains also proteases, digesting protein. The role of these proteases is yet unknown. And breast milk does not contain intact cow milk protein, but contains cow milk peptides. The digestion of partially hydrolysed protein may result in different metabolites than intact protein. Whether this is clinically relevant or not, is yet unknown. Overall, it is the opinion of the author that a partially hydrolysed protein may be considered as an option as protein source of a starter formula for every non-exclusively breastfed infants. It then becomes a cost/benefit discussion, which is difficult because cost of formula does vary substantially from country to country [19].

## 4. Symptoms and Diagnosis

Symptoms related to cow milk intake develop usually within the first two months after its introduction and it is unusual for CMA to develop in a child older than one year of age [21]. Symptoms can be separated in IgE and non-IgE mediated, and according to literature the distribution can be estimated fifty-fifty [21]. Many infants develop symptoms in two or more organ systems. Typical IgE mediated symptoms include urticaria, angioedema, vomiting, diarrhea and anaphylaxis. Dermatitis and rhinitis can be IgE and non-IgE mediated. Vomiting, constipation, hemosiderosis, malabsorption, villous atrophy, eosinophilic proctocolitis, enterocolitis and eosinophilic esophagitis are non-IgE mediated reactions. In addition, respiratory symptoms such as chronic rhinitis and asthma may be caused by CMA [22]. Irritability, fuzziness and colic are sometimes the only symptoms of CMA [3,23]. Whether diagnostic investigations such as IgE, specific RAST and skin prick tests should be performed depends on local facilities and routines, but they are not routinely recommended in the guidelines [3,21]. Total IgE is not helpful in the diagnosis of CMA, but the IgE level is related to the development of tolerance: the lower the total IgE, the more rapidly tolerance develops [21]. Specific IgE and skin prick tests may contribute to confirm the suspected diagnosis, although false positive results do exist. The atopy patch test, which is popular in France, has not been considered as a recommended diagnostic test in guidelines [3,21]. Negative test results do not exclude allergy [3]. Other diagnostic tests are only possible in specialized laboratories or indicated in very distinct clinical conditions, such as mucosal biopsies in infants presenting with blood in their stools. There is no place for the (expensive) determination of IgG4-antibody levels as these are considered to demonstrate contact of the immune system with the antigens but do not suggest an allergic reaction [3,21,23]. 

A symptom-based score, the Cow Milk Related Symptom Score (CoMiSS^TM^) has recently been developed to raise awareness of symptoms related to the ingestion of cow milk [24]. A challenge test is likely to be positive in 80% of patients if an initial score of more than 12 decreases to less than half with an eHF [25]. Therefore, it is hoped that, when it is validated, the CoMiSS^TM^ may become a valuable diagnostic tool [24]. 

The majority of the guidelines accept an open challenge in infants suspected of CMA, although a double-blind challenge test is considered to be the gold standard for diagnosing CMA [3,21,23]. Standardized procedures on how to perform a challenge test have been published (Table 2) [3,21,23]. A challenge test should always be performed under medical supervision, but it does not have to be systematically performed in a hospital environment. Hospitalization is recommended if it is suspected that acute, severe or unpredictable symptoms could occur [3]. Parents are often reluctant to perform a challenge test, because it will make the allergic child sick again. In addition, the results of a challenge are often difficult to interpret. While immediate reactions are relatively easy to pick up, delayed reactions are more difficult to detect. A group of experts published a standardized double-blind placebo-controlled food challenge [24]. This certainly has the merit to be scientifically sound but has the disadvantage to be difficult to apply in daily practice in not experienced centers or at primary health care level. Specifically for a cow's milk challenge, European experts have proposed an open prolonged challenge: after a half day challenge under medical supervision, the patient returns home and parents need to continue the challenge by providing a sufficient daily intake of at least 200 mL of milk per day [3,21]. Indeed, about half of the children will develop a delayed reaction, which will only be picked up if the parents are collaborating and the follow up is adequate. Double-blind challenge tests cover only the first part of the challenge test, which is under medical supervision.

If negative, the infant should drink at least 200 m of cows’ milk-based infant formula each day for the next 2 weeks and the parents should be contacted daily by a healthcare professional or should contact a healthcare professional if symptoms occur so that a late reaction can be documented.

## 5. Treatment

The vast majority of infants with suspected CMA will be formula-fed and present with a combination of the symptoms listed in the CoMiSS™ [24]. Guidelines recommend an elimination diet with a whey or casein-based eHF with clinical proof of efficacy for two to four weeks as the first option [3,23] (Table 3). The CoMiSS™ score can contribute to quantify clinical improvement. Hydrolysates strengthen the epithelial barrier, modulate T-cell differentiation and decrease inflammation [26]. Some studies suggest a role for hydrolysates in manipulating pathogen recognition receptors signaling as underlying mechanism. Peptides from hydrolysates have been shown to bind to TLR2 and TLR4 and influence cytokine production in epithelial cells and macrophages. Current insight suggests that hydrolysates may actively participate in modulating the immune responses in subjects with and those at risk to develop CMA [26]. If the symptoms do not improve, then CMA is unlikely. The percentage of patients tolerating the eHF will depend on the selection of patients. In eHFs, most of the nitrogen is present as free amino acids and peptides <1500 kDa [27]. During CMA treatment, allergenic peptides may be potentially harmful. Therefore, peptides that have reduced allergenicity but are capable to induce tolerance are recommended. The World Allergy Organization Diagnosis and Rationale for Action against Cow’s Milk Allergy (WAO-DRACMA) guidelines recommend cow milk based eHF over soy infant formula in IgE-mediated CMA [10]. Amino acid based formula (AAF) is recommended if formula-fed infants present with the rare condition of anaphylaxis and in eosinophilic esophagitis, or when the child does not tolerate to the eHF and CMA is a likely diagnosis (because failure of eHF has been reported) or when the cost/benefit analysis is in favor of the AAF [3,21]. However, eHFs have been reported to be effective in adults with eosinophilic esophagitis caused by cow milk [28]. If a strict AAF diet does not result in an improvement of the symptoms, the patient does not suffer CMA. In case of anaphylaxis, the long-term management of such infants should include a challenge with an eHF before cow milk is (re-)introduced. This should be carried out after 6 to 9 months or when the infant is one year old and always in a hospital environment [3,23].

If an eHF is not available, if the infant refuses to drink it or if it is too expensive, a rice hydrolysate or a soy infant formula are considered as second choices. Since pHF has longer peptides than eHF, pHF may trigger symptoms in sensitized infants (3,10,23). Therefore, a cow milk based pHF is not suitable for treating CMA. While eHF needs to be tolerated by >90% of patients, pHF will be tolerated by less than half of infants with CMA [3]. 

Thickened eHF and AAF are commercially available [30,31] to treat simultaneously CMA and infant regurgitation. Whether an eHF is thickened or not seems not to be relevant in CMA; however, when the challenge test is negative, the thickened eHF is more effective in reducing regurgitation than the non-thickened [30]. Up to now allergic reactions to the thickening agent in these formulas have not been reported. 

Partial and extensively rice hydrolyzed formula are commercialized and, in some parts of the world, soy (hydrolyzed) infant formula also exists. Since rice hydrolysates are relatively new, they are not (yet) considered in published guidelines. The clinical efficacy of rice hydrolysates, partial and extensive, seems excellent [32,33]. Rice hydrolysates are free of CMP allergens. Rice hydrolysates are less expensive than cow milk based eHF. The content of arsenic in rice may be a safety issue limiting the use of rice. There is an FDA warning against the use of rice in infants and young children regarding rice feed thickeners and rice cereals. Therefore the arsenic content in rice based infant formula should be determined and declared on the label [34]. The arsenic content in infant formula is reported to be within the safety limits. Other mammalian milks such as sheep milk and goat milk are not indicated in the treatment of CMA [3]. Infant formulas based on goat milk are on the market in a substantial number of countries, but the high incidence of cross-reactivity in CMA patients results in the fact that they cannot be recommended for infants with CMA [35]. Significant cross-sensitization to milk proteins derived from kosher animals exist in patients allergic to CMP, but far less so than the milk proteins tested from non-kosher animals [35,36]. Camel and mare milk have not been evaluated as possible options [37,38,39], but ass milk in particular has been shown to be effective in treating CMA [40,41]. The DRACMA guidelines even recommend donkeys milk as third option in constipation due to CMA. However, none of these alternative options fulfill the nutritional and compositional requirements for infant formula and as a result they cannot be recommended in the treatment of CMA. Consumption of unprocessed cow milk in young infants protects against respiratory infections [42]. However, unprocessed cow milk can as well not be recommended in infants for nutritional reasons. The epitopes in raw, cooked or baked milk differ [43] as baked milk was reported to be tolerated in patients with eosinophilic esophagitis as presentation of CMA [43]. Although use of hypoallergenic baked milk in oral immune therapy is a promising therapy, care must be taken before its administration in baked milk-reactive patients because of the risk for anaphylaxis and only limited increase in challenge threshold attained [44].

Soy infant formula has existed for longer than one century, but its popularity varies greatly [23]. The Agence Française de Sécurité Sanitaire des Aliments drew attention to the presence of isoflavones and their unknown impact on infant health. Isoflavones have been shown to induce estradiol-like effects in animal models [45]. The American Academy of Pediatrics reviewed the literature and summarized that 10% to 14% of infants with CMA will become soy-sensitized, with a higher incidence in non-IgE mediated CMA than in IgE mediated CMA [46]. According to a recent meta-analysis, the prevalence of soy allergy was 0.5% in the general population, but the prevalence of sensitization after the use of soy infant formula was 8.7% [47]. Therefore, it seems logic today to recommend a clinically tested eHF as first option in the management of CMA, and to recommend rice hydrolysates as a second option and soy as third option. 

A lack of effective and approved treatment has led to strict avoidance of the culprit food proteins being the only standard of care [2]. Several food immunotherapies are being developed; these involve oral, sublingual, epicutaneous, or subcutaneous administration of small amounts of native or modified allergens to induce immune tolerance [2,48]. Oral immunotherapy is a promising but still experimental method to treat children with cow’s milk allergy [49]. The approach generally follows the same principles as immunotherapy of other allergic disorders and involves the administration of small increasing doses of food during an induction phase followed by a maintenance phase with regular intake of a maximum tolerated amount of food [50]. Most research has been conducted with oral immunotherapy due to its efficacious and relatively safe profile but remains an investigational treatment to be further studied before advancing into clinical practice [2,48]. Determination of IgE and IgG4 epitope binding may contribute to select candidates for oral immune therapy [51]. Oral immune therapy carries significant risk of allergic reactions [51]. The ability of oral immune therapy to desensitize patients to particular foods is well-documented, although the ability to induce tolerance has not been established [51]. Recent data suggest that oral immune therapy may induce long term tolerance in half of the children [52]. Markers of allergy such as blood eosinophils and serum IgE decreased and milk-specific IgG and IgG4 increased during oral immune therapy [49]. Adipokines, leptin and resistin, which functionally are cytokines linked to Th1-type response, increase during oral immune therapy [49]. The high frequency of allergic adverse reactions of the various approaches highlighted the need of refinements in the strategies. A careful review of the patients who received food oral immune therapy in controlled trials confirmed that adverse events were not rare but that ~90% of children could achieve an effective desensitization [53]. A promising strategy for preventing IgE cross-linking and thus enhancing safety of immune therapy, while still activating T cells, is the use of tolerogenic peptides [2]. Additional bigger, multicentric and randomized-controlled studies must answer multiple questions including optimal dose, ideal duration of immunotherapy, degree of protection, efficacy for different ages, severity and type of food allergy responsive to treatment [48]. The procedure remains investigational and should be performed only by trained physicians, especially in the pediatric setting [53]. Immunotherapy for food allergy is still not ready for the clinic, but current and upcoming studies are dedicated to collect enough evidence for the possible implementation of allergen-SIT as a standard treatment for food allergy [2].

## 6. Gut Microbiota

The role of the GI microbiota in food allergy has been a topic of major interest since many years. Oral tolerance is the consequence of a systemic absence of a response to dietary antigens. Early infancy is a window during which gut microbiota may shape food allergy outcomes in childhood [54]. Dietary antigens and intestinal microbiota are known to make up the majority of the antigen load in the intestine. The GI microbiome plays a strong role in the orientation of the immune response [55]. 

Food allergy is associated with alterations in the gut microbiota or dysbiosis early in life that may be predictive of disease persistence versus tolerance acquisition [56]. Qualitative and quantitative differences in the composition of the gut microbiota between infants who will and infants who will not develop allergy are demonstrable before the development of any clinical manifestations of atopy [57,58]. Gut microbiome composition at age 3 to 6 months was associated with acquisition of tolerance to milk proteins by age 8 years, with enrichment of Clostridia and Firmicutes in the infant gut microbiome of subjects with resolved CMA [54]. Metagenome functional prediction supported decreased fatty acid metabolism in the gut microbiome of subjects whose CMA resolved [54]. As a consequence, bacterial taxa within Clostridia and Firmicutes could be studied as probiotic candidates for milk allergy therapy [54]. Data obtained in murine models of food allergy suggest that microbial therapy with protolerogenic bacteria such as certain Clostridial species holds promise in future applications for prevention or therapy of food allergy [59]. Extrapolation from in vitro data suggests that supplementing infant formulas such as eHF with prebiotics or probiotics (*Lactobacillus* (L.) *rhamnosus* GG, *Bifidobacteria* (B.) breve) may offer an additional benefit [60].

### 6.1. Prebiotics

Dietary supplementation with short chain galacto-oligosaccharides (scGOS), long chain fructo-olgosaccharides (lcFOS) and/or pectin-derived acidic oligosaccharides during sensitization effectively reduce allergic symptoms but differentially affect mucosal immune activation in whey-sensitized mice [61]. A beneficial effect of prebiotics on the development of atopic dermatitis in a high risk population of infants was shown for the first time in this paper [62]. Although the mechanism of this effect requires further investigation, it appears likely that oligosaccharides modulate postnatal immune development by altering bowel flora and have a potential role in primary allergy prevention during infancy [63]. These findings were confirmed by demonstrating that early dietary intervention with oligosaccharide prebiotics has a protective effect against both allergic manifestations and infections [64]. Later, this effect was also shown in non-at-risk infants [63]. The observed dual protection lasting beyond the intervention period, up to the age of five years, suggests that an immune modulating effect through the intestinal flora modification may be the principal mechanism of action [60,63]. This mechanism has now been demonstrated [64]. 

The addition of lactose to an eHF is able to positively modulate the composition of gut microbiota by increasing the total fecal counts of L/B and decreasing that of Bacteroides/Clostridia [65]. The positive effect is completed by the increase of median concentration of short chain fatty acids, especially for acetic and butyric acids demonstrated by the metabolomic analysis [66]. However, the ESPGHAN Committee on Nutrition concluded in 2011 that there was insufficient evidence to recommend the use of prebiotics in infant formula to prevent atopic disease [67]. But, based on GRADE evidence to decision frameworks, the WAO guideline panel suggests using prebiotic supplementation in not-exclusively breastfed infants and not using prebiotic supplementation in exclusively breastfed infants [68]. Both recommendations are conditional and based on very low certainty of the evidence [68].

Human milk oligosaccharides (HMOs) are a group of complex sugars that are highly abundant in human milk, but currently not present in infant formula. Literature indicating that HMOs play a major beneficial and facilitating role in the development of the infant’s microbiome and thus immune development is abundant and unequivocal. However, there are over 100 different HMOs, with specific properties and functions. HMOs are not digested by the infant and serve as metabolic substrates for select microbes, contributing to shape the infant gut microbiome. HMOs provide a main substrate to help shape the infant’s gut microbiota and affect the maturation of the intestinal mucosal immune system [69]. Higher HMO diversity at the age of one month was associated with lower total and percentage fat mass [69]. At the age of 6 months, each 1-μg/mL increase in lacto-N-fucopentaose was associated with a 1.11-kg lower weight and a 0.85-g lower lean mass [69]. These findings support the hypothesis that differences in HMO composition in mother's milk are associated with infant growth and body composition [69].

HMOs act as soluble decoy receptors that block the attachment of viral, bacterial or protozoan parasite pathogens to epithelial cell surface sugars, which may help prevent infectious diseases in the gut and also the respiratory and urinary tracts. HMOs alter host epithelial and immune cell responses. Secretor milk contains higher concentrations of total and fucosylated HMOs than does nonsecretor milk. These HMO concentrations can be correlated to the health of breastfed infants in order to investigate the protective effects of milk components [70]. HMOs have the potential to selectively enrich the beneficial intestinal microbiota in breast-fed infants. Infants that received human milk with low Lacto-N-fucopentaose III concentrations were more likely to become affected with cow’s milk allergy when compared to high LNFP III-containing milk (odds ratio 6.7, 95% CI 2.0–22) [71].

Up to now, only a limited number of HMOs have been synthetized and studied in infant formula, showing beneficial results. It is however unclear if a single HMO is more beneficial for the infant's immune system development than the artificial prebiotic oligosaccharides such as galacto- and fructo-oligosaccharides. 

### 6.2. Probiotics

The administration of probiotics may contribute to the restoration of the healthy equilibrium of the GI microbiota and contribute to the efficacy of an elimination diet in CMA. Probiotics are known to cross-talk with the intestinal immune cells. Probiotic bacteria have different modes of action in the intestinal lumen: they hydrolyze peptides that are potentially antigenic to non-antigenic peptides; they decrease the intestinal permeability and, as a consequence reduce the penetration of antigens from the gut lumen to the systemic circulation; they stimulate the local production of IgA and they regulate local inflammatory responses and stimulate the differentiation and growth of the GI mucosa [41]. Administration of L GG to children under the age of 2 years suffering from eczema and with a challenge-proven food allergy has been shown to result in a significant decrease in the eczema score [72]. A formula supplemented with L GG also decreased GI symptoms in infants with eczema [73]. A cow milk challenge in allergic infants resulted in an increase of fecal IgA levels and a decrease of the TNF-α level compared to a placebo [74]. L. GG has been shown to substantially increase the memory B cells and stimulate interferon-γ secretion in infants with CMA and with IgE-associated dermatitis, but not in healthy infants [75]. These findings support the hypothesis that infants with an atopic predisposition may have an aberrant pattern of intestinal microbiota and this explains why the beneficial effects of probiotics are only seen in this group [76]. In infants with colitis, supplementing a casein eHF with L. GG significantly enhanced the recovery of the inflammation in the colonic mucosa in comparison to the same hydrolysate without the probiotics [76]. In the group that received the probiotic, fecal calprotectin and the number of infants with ongoing occult blood in stools after one month were significantly smaller [77]. The primary goal in the treatment of CMA is, of course, for the symptoms to disappear. However, the second, and almost equally important objective, is to acquire oral tolerance. As an eHF supplemented with L casei CRL431 and B lactis BB-12 failed to accelerate tolerance, this effect may be strain specific [78]. In a trial that compared an eHF without and with L GG, a double-blind placebo-controlled food challenge (at least 1.4 × 10^7^ CFU/100 mL) was negative in 15/28 (53.6%) infants without L. GG and in 22/27 (81.5%, *p* = 0.027) with L. GG. These findings may prove innovative in the therapeutic approach to treating infants with CMA by accelerating the acquisition of tolerance [79]. 

Consumption of probiotic milk products was related to a reduced incidence of atopic eczema and rhinoconjunctivitis, but not associated to the incidence of asthma by 36 months of age [80].

Most tolerant infants showed a significant increase in fecal butyrate levels, and those taxa that were significantly enriched in these samples [81], exhibited specific strain-level demarcations between tolerant and allergic infants. Data suggest that a casein eHF with L. GG promotes tolerance in infants with CMA, in part, by influencing the strain-level bacterial community structure of the infant gut [81].

Perinatal probiotic administration is safe in long-term follow-up [82]. Children receiving L. rhamnosus GG perinatally tended to have decreased allergy prevalence [82]. The subgroup analysis based on the type of treatment suggested that both L. alone and L. with B. are protective against atopic dermatitis (OR = 0.70, *p* = 0.004; OR = 0.62, *p* < 0.001). Probiotics seem to have a protective role in atopic dermatitis prevention if these are administered during the pre- and postnatal period in both general and allergic risk populations [82]. However, the ESPGHAN Committee on Nutrition concluded in 2011 that there was insufficient evidence to recommend probiotics to prevent atopic disease [67]. However, considering all critical outcomes in this context, the WAO guideline panel determined that there is a likely net benefit from using probiotics resulting primarily from prevention of eczema. The WAO guideline panel suggests: (i) using probiotics in pregnant women at high risk for having an allergic child; (ii) using probiotics in women who breastfeed infants at high risk of developing allergy; and (iii) using probiotics in infants at high risk of developing allergy [83]. Probiotic compounds may contain hidden allergens of food and may not be safe for subjects with allergy to cow milk or hen’s egg [84,85].

Post-sensitization administration of non-digestible oligosaccharides and *Bifidobacterium breve* M-16 V were shown to reduce allergic symptoms in mice [86]. Studies demonstrate that an AAF with synbiotics is safe and well tolerated and promotes normal growth when fed to healthy full-term infants as the sole source of nutrition and is hypoallergenic in subjects with CMA [87]. 

## 7. Conclusions

The diagnosis of CMA is still a challenge. Cow milk based eHF remains the recommended and preferred therapeutic choice, while AAF is reserved for the most severe cases. Rice hydrolysates and soy informant formulas are second choice options. Manipulation of the gut microbiotica may enhance the development of oral tolerance. Hydrolysates, in particular pHF with proven efficacy, may become a protein source in starter infant formula. Since the efficacy of hydrolysates in the prevention of allergic disease is debated, some guidelines recommend these formulas in infants at risk for atopic disease, while other meta-analyses and some countries do not recommend the use of these formulas in prevention. However, it is obvious that these formulas do not harm. Similar, although the clinical evidence for a benefit of additional prebiotics or HMOs and/or probiotics is limited, supplementation of hydrolysates should be considered as adverse effects have not been reported.

## Figures and Tables

**Table 1:** Symptoms and signs related to CMA.

**General**
Anaphylaxis Food protein induced enterocolitis syndrome (FPIES; shock-like symptoms with severe metabolic acidosis, vomiting and diarrhea)
**Gastro-Intestinal**
Failure to thrive, anorexia, refusal to feed, early satiety Dysphagia, dyspepsia Abdominal pain, colic Nausea, regurgitation, emesis Diarrhea with or without protein loss or bleeding Constipation with or without perianal rash Iron-defeiciency anemia due to occult blood loss
**Respiratory**
Respiratory distress Runny nose, chronic coughing Wheezing/stridor
**Dermatological**
Urticaria, atopic eczema, angioedema.

**Table 2:** Example of standardized protocol for open challenge test.

Drop of formula on the lips If there is no reaction after 15 min, the formula is given orally and the dose is increased stepwise (0.5, 1, 3, 10, 30, 50 to 100 mL) every 30 min Additional observation for at least 2 h

**Table 3:** Recommended therapeutic options according to different guidelines for different symptoms and signs of cow's milk allergy.

	Australia [29]		Dracma [10]		Espghan [3]	
	1st choice	2nd choice	1st choice	2nd choice	1st choice	2nd choice
GI syndromes	eHF soy (if >6 months)	AAF eHF	eHF	AAF	eHF	AAF
proctocolitis	eHF	AAF			eHF	AAF
Eos Eso	AAF		AAF		AAF	
Immediate FA	eHF soy (if >6 months)	AAF eHF	eHF	AAF/Soy	eHF	AAF
FPIES	eHF	AAF	eHF	AAF	eHF	AAF
Atopic eczema	eHF soy	AAF eHF	eHF	AAF/Soy	eHF	AAF
urticaria			eHF	AAF/Soy	eHF	AAF
Constipation			eHF	AAF		
Heiner syndrome			AAF	eHF		
